# Elevated CA 125 in a CASE of Leaking Endometrioma

**DOI:** 10.1155/2018/2385048

**Published:** 2018-09-12

**Authors:** Svetha Rao, Supuni Kapurubandara, Anbu Anpalagan

**Affiliations:** ^1^Liverpool Hospital, Australia; ^2^Westmead Hospital, Australia

## Abstract

Extremely elevated CA 125, usually suggestive of ovarian malignancy, can be found in physiological or benign conditions such as endometriosis. We present a case of an extremely elevated serum CA 125 level in a patient with stage four endometriosis and bilateral unruptured ovarian endometriomas, with evidence of leakage unilaterally. To avoid costly and unnecessarily invasive tests and procedures it is important to consider the differential diagnosis of endometriosis and/or leaking endometrioma in patients with a profoundly elevated CA 125 level.

## 1. Introduction

The cancer antigen (CA) 125 is a high molecular weight glycoprotein, which originates from coelomic epithelium, which is expressed by normal tissues such as the endometrium, peritoneum, pericardium, and epithelial ovarian carcinomas (EOCs) [1]. It is most commonly used as a biomarker for EOC for the purposes of diagnosis, monitoring of disease progression, and response to treatment [2]. CA 125 also has an important role in differentiating benign and malignant pelvic masses, especially preoperatively, as higher CA 125 levels are considered to correlate with a higher probability of malignancy [2]. However, serum CA 125 levels can be elevated in other malignancies as well as various physiological and benign conditions such as endometriosis, uterine fibroids, pelvic inflammatory disease, early pregnancy, and normal menstruation [2, 3]. The positive predictive value of CA 125 for ovarian cancer is high among postmenopausal women (96%) [4] but is associated with a lower specificity among premenopausal women given the various benign conditions that can lead to elevated CA 125 levels [2]. Therefore, benign conditions such as endometriosis should be considered as differential diagnoses in the context of an elevated CA 125 level, especially among premenopausal women. Profoundly raised CA 125 in the absence of malignancy is rare; we review the literature regarding similar cases to help guide assessment and management of such patients.

## 2. Case Presentation

A 27-year-old nulliparous woman presented to the emergency department complaining of abdominal pain on the background of chronic pelvic pain.

On admission, an enlarged right ovary 150cc in volume with a cyst measuring 6.5cm and low internal echoes was demonstrated on pelvic ultrasound. Abdominopelvic computed tomography (CT) scan also demonstrated a 6.5cm dense right ovarian cyst with a moderate volume of free fluid and no evidence of appendicitis. Tumour markers taken at the time of acute presentation demonstrated a serum CA 125 level of 8142 U/ml (reference range: <35 U/ml) which had significantly increased from 115 U/ml when performed 12 months prior. Serum alpha fetoprotein (AFP) and human chorionic gonadotropin (hCG) levels were both <2 U/ml.

She was referred to the gynaecology clinic at Westmead hospital for further urgent review and management. An ultrasound scan for deep infiltrating endometriosis (DIE) verified the presence of a right ovarian cyst (6.3 x 5.0 x 4.4cm) with bowel adherent to the posterior aspect of the uterus. A gynaecological oncological opinion was sought at this time in light of the significantly raised CA 125 recommending a repeat level in 2 weeks on the provisional diagnosis of endometriosis after reviewing the ultrasound images and patients history of initial presentation. Repeat measurement of serum CA 125 level taken two weeks from her initial presentation demonstrated a lower but still significantly elevated level of 2038 U/ml (day 12). Serum carcinoembryonic antigen (CEA) and CA 19.9 were <2 U/ml and 430 U/ml (reference range: <37 U/ml), respectively.

A multidisciplinary discussion with a gynaecologist oncologist was conducted to determine further management. Based on the images and decreasing serum CA 125 level an endometriotic leak from an ovarian endometrioma was considered most likely, with ovarian malignancy being the main differential and unlikely diagnosis.

**Figure 1:**
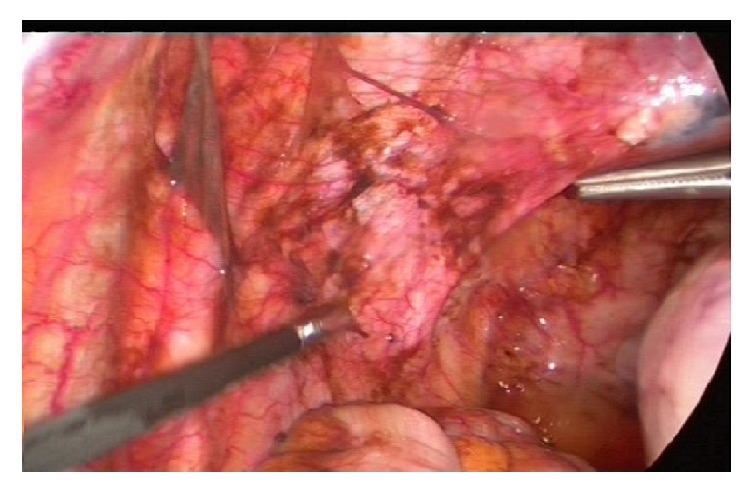
**Intraoperative images of widespread endometriotic deposits**, suggestive of leaking endometrioma.

**Figure 2:**
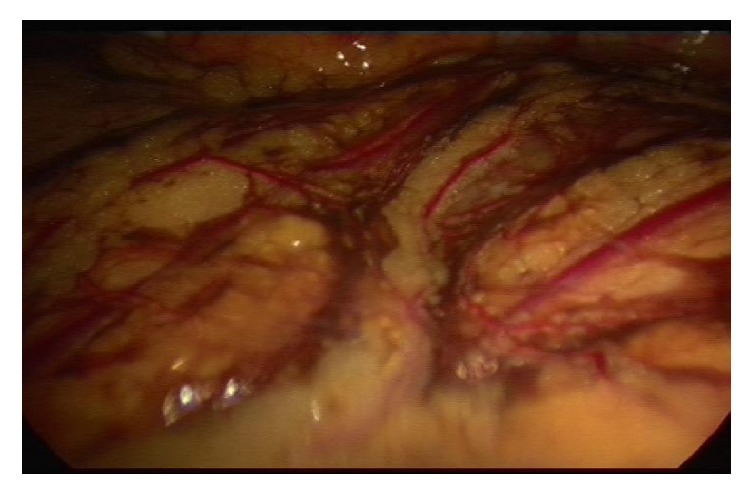
**Intraoperative images of widespread endometriotic deposits**, suggestive of leaking endometrioma.

At laparoscopy on day 58, stage four endometriosis and bilateral unruptured ovarian endometriomas, with features suggestive of leakage unilaterally, were revealed. Widespread endometriotic deposits were found at the upper and anterior abdominal wall, omentum, and bilateral uterosacral ligaments, likely secondary to leaking endometrioma (Figures 1 and 2). Laparoscopic excision of endometriosis, bilateral ureterolysis, bilateral excision of endometrioma, and insertion of a Mirena^©^ intrauterine device were performed.

Histopathological examination confirmed the diagnosis of endometrioma. The patient recovered uneventfully and was discharged on the third postoperative day. At the third postoperative week the patient remained in a stable condition and routine follow-up with the general practitioner was recommended.

## 3. Discussion

CA 125 was first identified as an ovarian cancer antigen in 1981 [19] and later developed as a biomarker for EOC when serum levels > 35 U/ml were found in over 80% of patients with EOC but only 1% of healthy women [4]. The positive predictive value of CA 125 (> 95 IU/ml) for ovarian cancer is high among postmenopausal women (96%) [4] and is associated with a high sensitivity and specificity, of 69-97% and 81-93%, respectively [20]. While most commonly used in EOC, serum CA 125 levels can be elevated in other malignancies and various physiological and benign conditions including endometriosis [2, 3]. Consequently, CA 125 is associated with a poorer specificity among premenopausal women given the various benign conditions that can lead to an elevated CA 125 level [2, 21] and a physiologic serum half-life of approximately 6 days [22]. Routine CA 125 level is therefore not recommended in all premenopausal women with a simple appearing ovarian cyst [23].

Patients with endometriosis often do not have CA 125 levels > 100 U/ml [13, 16]. Still, endometriosis is one of the most common benign conditions associated with elevated serum CA 125 [14]. CA 125 has been extensively studied as a biomarker for endometriosis, with two meta-analyses concluding it has limited utility as a diagnostic marker for endometriosis given its low sensitivity (20-50%) [1, 24, 25]. This is supported by international guidelines for endometriosis which do not recommend the measurement of serum CA 125 level as part of routine diagnostic work-up [26]. The relationship between elevated CA 125 levels and endometriosis has been well established in the literature, with levels reflecting both the severity and the progression of the disease [3, 27, 28]. Elevated serum CA 125 levels are often related to ovarian endometriomas and endometriosis of higher severity such as stages three and four [3, 14, 23, 29]. CA 125 levels have also been shown to decrease following both medical and surgical treatment of endometriosis [10, 29].

Extremely elevated CA 125 levels have been reported in the presence of both ruptured [5, 7, 9, 10, 12, 30] and unruptured endometriomas [13–18] (Tables 1 and 2). The highest CA 125 level reported in proven endometriosis is 9537 U/ml following acute rupture of an endometrioma [7]. In the present case, we report an extremely elevated serum CA 125 level of 8142 U/ml in a patient found to have stage four endometriosis, bilateral unruptured ovarian endometriomas with evidence of leakage unilaterally, and widespread endometriotic deposits when viewed intraoperatively. The previous highest CA 125 level in the context of an unruptured endometrioma was 7900 U/ml [16]. This carries significant clinical importance as an elevated CA 125, especially in the presence of a pelvic mass, can mimic and raise suspicion of a malignant process leading to unnecessarily invasive procedures [15, 30]. Education of the patient regarding the significance of an elevated ***tumour*** marker in the absence of a malignant tumour is of prime importance, with sensitivity to the potential emotional distress such words can impose. The decision to manage as a benign gynaecological condition compared to a potentially malignant case should be discussed.

There are multiple theories behind elevated serum CA 125 levels in endometriosis. The fluid within an endometriotic cyst [or endometrioma] is thought to be rich in CA 125 with concentrations reported to be > 10000 U/ml [31]. Following leakage of endometriotic fluid, from an endometrioma, this fluid will subsequently cover peritoneal surfaces which may be absorbed into the peripheral circulation and cause peritoneal inflammation, resulting in an elevated CA 125 level [7, 12, 16, 32, 33]. Given the increase in peritoneal fluid in the presence of mild endometriosis and the presence of higher levels of CA 125 in peritoneal fluid compared to corresponding serum levels [34], this could also contribute to elevated serum CA 125 measurements.

The reason for high CA 125 concentrations in cyst fluid compared to serum levels is attributed to the thick wall of the endometriotic cyst preventing large CA 125 glycoprotein molecules from diffusing out of the cyst and reaching systemic circulation; however, this inhibition of CA 125 molecules is not believed to be absolute [14, 35]. Elevated serum CA 125 levels in endometriosis are also attributed to a higher surface area of endometrial tissue such as endometriotic cysts [9], deep infiltrating endometriotic nodules, adhesions [25], and the stage of disease [13]. In our case leakage of CA 125 rich cystic fluid into the peritoneal cavity in combination with stage four endometriosis and bilateral endometriomas could explain the extremely elevated serum CA 125 level of 8142 U/ml, which had dramatically rose from 115 U/ml one year prior as well as the subsequent level of 2038 U/ml two weeks later. Concurrent menstruation has been shown to cause up to three-fold increase in CA 125 level among women with endometriosis [28]. While this could have impacted the initial level in this case, it does not account for a persistently elevated CA 125 two weeks later.

While there have been several reports of elevated serum CA 125 levels in both ruptured and unruptured endometriomas, the present case reports a rare finding of an extremely elevated serum CA 125 level in the context of bilateral endometriomas, with evidence of leakage unilaterally. This case demonstrates that serum CA 125 levels can be extremely elevated due to an unruptured leaking endometrioma and highlights the importance of considering the differential diagnosis of endometriosis and or endometrioma in patients with an elevated CA 125, when suspecting ovarian carcinoma as the cause of an adnexal mass.

## Figures and Tables

**Table 1:** Summary of cases of ruptured endometrioma with elevated CA 125 levels†.

**Author & Year**	**Type of study and number of patients (n)**	**CA-125 level (IU/ml)**	**Clinical presentation**	**Imaging findings**	**Operative intervention / Management**
Johansson J et al. (1998) [5]	Case report n = 1	9300	Abdominal pain	USS: Rt homogeneous ovarian 7x10cm	Laparotomy, Excision of endometrioma

Kashyap RJ. (1999) [6]	Case report n = 1	6114	Abdominal pain, Nausea	USS: 11 cm complex cyst	Laparotomy, Rt oophorectomy

Kurata, H et al. (2002) [7]	Case report n = 1	9537	Abdominal pain	USS: homogenous bilateral ovarian cysts, FF in pelvis MRI: ovarian masses, bloody liquid with T1- high signal and T2-low signal	Laparoscopy, enucelation of cysts

Cengiz et al. (2012) [8]	Case report n = 1	174.87	Abdominal pain, Nausea	USS: Lt heterogenous adnexal mass 6x8cm, FF in pelvis	Laparoscopy, Enucleation of cyst

A.K. Rani et al. (2012) [9]	Case report n = 1	9391	Abdominal pain	USS: Rt homogenous adnexal mass 10.5x7cm, moderate ascites CT: b/l adnexal mass, minimal ascites, nil lymphadenopathy	Laparotomy, excision of endometrioma

Duran M et al. (2013) [10]	Case report n = 1	2556	Pelvic and Abdominal pain, Dysuria	USS & CT: Lt heterogenous adnexal mass 5x5cm	Laparoscopy, Excision of endometrioma

Dereli et al. (2014) [11]	Case report n = 1	143.72	Bilateral pelvic masses	USS: hypoechoic bilateral adnexal masses	Laparoscopy, Rt adnexectomy, Lt cystectomy

X. Dai et al. (2015) [12]	Retrospective cohort n = 43	797.89 ± 1106.52	Abdominal pain, Pelvic mass, Asymptomatic	-	Laparoscopy/ Laparotomy

USS: ultrasound; CT: computerised tomography; MRI: magnetic resonance imaging; FF: free fluid; PoD: pouch of Douglas.

†In all cases reviewed endometrioma was confirmed histologically.

**Table 2:** Summary of cases of unruptured endometrioma with elevated CA 125 levels†.

**Author & Year**	**Type of study and number of patients [n]**	**CA-125 level [IU/ml]**	**Clinical presentation**	**Imaging findings**	**Operative intervention / Management**
Yilmazer M et al. (2003) [13]	Case report n = 1	1741.8	Abdominal pain, Bilateral adnexal masses	USS & CT: bilateral adnexal cystic masses	Laparoscopy, B/L cystectomy

Shiau C-S et al. (2003) [14]	Case report n = 1	6310	Pelvic mass, Abdominal pain, Nausea	USS & CT: homogenous Lt adnexal cystic mass 75mm	Laparotomy, Enucleation & excision of cyst

Atabekoglu C et al. (2003) [15]	Case report n = 1	3890	Abdominal pain, Dysmenorrhea	CT: right cystic ovarian mass of 12x10 cm	Laparotomy, Rt adnexectomy

Kahraman K et al. (2007) [16]	Case report n = 1	7900	Adnexal mass	USS & MRI: homogeneous Lt adnexal cystic mass	Laparoscopy, Cystectomy, U/L salpingectomy

Hosseini, M et al. (2009) [17]	Case report n = 1	2000	Abdominal pain, Dysmenorrhea	USS: bilateral ovarian cystic masses	Laparotomy, B/L cystectomy

Peker N et al. (2013) [18]	Case report n = 1	1061	Pelvic mass	USS: homogenous left ovarian cystic mass, FF at PoD	Laparotomy, Enucleation of cyst

USS: ultrasound; CT: computerised tomography; MRI: magnetic resonance imaging; FF: free fluid; PoD: pouch of Douglas

†In all cases reviewed endometrioma was confirmed histologically.
